# Dynamic Compression Mechanical Properties of Polyoxymethylene-Fiber-Reinforced Concrete

**DOI:** 10.3390/ma15217784

**Published:** 2022-11-04

**Authors:** Luxin Guo, Rongxin Guo, Yong Yan, Yubo Zhang, Zhenhui Wang, Yifan Mu

**Affiliations:** 1 Yunnan Key Laboratory of Disaster Reduction in Civil Engineering, Faculty of Civil Engineering and Mechanics, Kunming University of Science and Technology, Kunming 650500, China; 2 School of Architectural Engineering, Yunnan Agricultural University, Kunming 650201, China

**Keywords:** concrete, polyoxymethylene fiber, dynamic compression mechanical properties, strain rate effect, constitutive model

## Abstract

The excellent overall performance of polyoxymethylene (POM) fiber enables it to show great potential for engineering applications. The effect of POM fibers on the dynamic compression mechanical properties of concrete is an important issue for its application in engineering structures such as airport pavement and bridges. It is necessary to investigate the dynamic compressive mechanical properties of POM-fiber-reinforced concrete (PFRC) under impact loading. The PFRC specimens with various POM fiber lengths (6, 8, 12, 16, and 24 mm) and ordinary-performance concrete (OPC) specimens were tested by utilizing the split Hopkinson pressure bar (SHPB). We studied the effect of fiber length and strain rate on the dynamic compression mechanical properties of PFRC and established a damage dynamic constitutive model for PFRC. The results indicate that the dynamic compressive strength, peak strain, ultimate strain, dynamic peak toughness, dynamic ultimate toughness, and dynamic increase factor (DIF) of the PFRC increased obviously with the increase in strain rate. POM fiber was found to be able to effectively improve the deformation ability and impact toughness of concrete. The dynamic compressive strength and impact toughness of PFRC with a fiber length of 8 mm was optimal at different strain rates. The POM fibers with 16 mm and 24 mm lengths negatively affected the dynamic compressive strength of the concrete. The fiber length variation had an insignificant effect on the DIF of PFRC. The established damage dynamic constitutive model for PFRC was fitted and analyzed, and it was found that the model is able to describe the dynamic characteristics of PFRC well. This study can extend POM fibers to engineering structures that may be subjected to impact loading and act as a reference for the design of PFRC under impact loading.

## 1. Introduction

Polyoxymethylene (POM) fiber is a new type of polymer fiber with high mechanical strength, excellent dimensional stability, and chemical corrosion resistance [1]. POM fiber has better dispersion and lower air entrainment than commonly used synthetic fibers such as polypropylene (PP) fiber, polyvinyl alcohol fiber (PVA) and polyethylene (PE) fiber, and POM fiber contains a large number of ether bonds in its molecular structure, resulting in higher interfacial bonding strength with inorganic materials [2,3]. POM fibers are more resistant to corrosion than steel fibers, which facilitates their service in harsh environments. Compared with other commonly used fibers, the application and research of POM fibers in the field of cementitious composite started late. However, due to the excellent comprehensive performance of POM fibers, they show great potential for engineering applications and are rapidly becoming a research hotspot.

In recent years, an increasing number of scholars have been conducting research on POM-fiber-reinforced cementitious composites. Yu et al. [3] have studied the effects of POM fibers and steel fibers on the mechanical properties of ultra-high-performance concrete (UHPC). It was shown that POM fibers significantly improved the flexural strength of UHPC; there was good adhesion between the surface of POM fibers and the matrix interface of the UHPC, and POM fiber had a positive effect on the fire resistance of UHPC. He et al. [1] found that POM fibers adversely affected the compressive strength of cementitious composites, but POM fibers significantly improved the flexural and uniaxial tensile properties of cementitious composites. Zhang et al. [4] conducted fiber/cement interface pullout and fiber fabric/cement interface pullout tests. They found that the bond strength of POM fibers to the cement interface was higher than that of PP fibers and polyethylene terephthalate (PET) fibers. Wang et al. [5] evaluated the effect of POM fibers on the flexural fatigue performance of airport pavement concrete and showed that the incorporation of POM fibers could effectively improve the bending fatigue deformation capacity of airport pavement concrete. POM fibers may reduce the fatigue life of airport pavement concrete.

**Figure 1:**
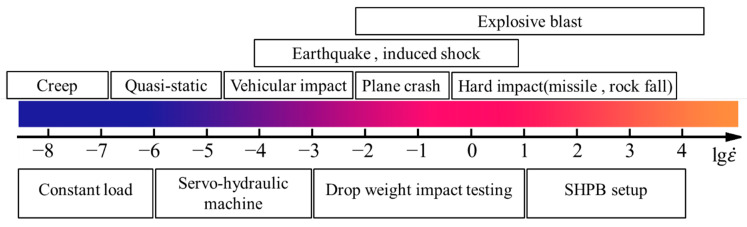
Typical strain rate range of concrete materials and the corresponding load cases.

The above shows that the current research on POM-fiber-reinforced cementitious composites mainly focused on quasi-static mechanical properties. However, the research literature on the dynamic mechanical properties and dynamic constitutive relationships of POM-fiber-reinforced concrete (PFRC) under impact loading is relatively scarce, and the dynamic mechanical properties of PFRC have not been studied enough. The excellent comprehensive performance of POM fibers makes their application valuable in engineering fields such as high-rise buildings, airport pavements, and bridges. These structures may be subjected to dynamic loads such as explosions, impacts, earthquakes, and tsunamis in increasingly complex service environments [6] (Figure 1 shows the typical strain rate range and corresponding loading of concrete materials [7,8]). In order to provide some reference for the design of PFRC under dynamic impact loads and to promote POM fibers for engineering structures that may be subjected to impact loads, such as airport pavements or bridges, to make the structures safer and reliable under dynamic loads, it is necessary to study the dynamic mechanical properties of PFRC.

Currently, the dynamic mechanical behavior of materials under high-strain-rate (10^1^–10^3^ s^−1^) loading is studied mainly by using the split Hopkinson pressure bar (SHPB) test setup [8]. Su et al. [9] used the SHPB setup to investigate the effects of micro steel fibers, twisted steel fibers, and the fiber aspect ratio and fiber volume fraction on the dynamic mechanical properties of UHPC. The study showed that UHPC reinforced by micro steel fibers had better dynamic strength when compared with the twisted-steel-fiber-reinforced UHPC. The dynamic strength of UHPC increased with the increase in fiber length. Liao et al. [10] used a 75mm-diameter SHPB setup to investigate steel-fiber-reinforced concrete’s dynamic compression mechanical properties with different fiber contents and aspect ratios. The results showed that when the fiber content was the same, the dynamic compressive strength of SFRC was the highest with a fiber aspect ratio of 50 at different strain rates. It can be seen from the above research that changes in the fiber length can affect the dynamic mechanical properties of concrete. The effect of POM fiber length variations on the dynamic compression mechanical properties of concrete is still unclear.

We used the 80 mm-diameter SHPB setup to conduct dynamic impact compression tests on ordinary-performance concrete (OPC) and five fiber lengths (6, 8, 12, 16, and 24 mm) of PFRC. The effects of the POM fiber length and strain rate on the failure pattern, dynamic compressive strength, deformation capacity, and impact toughness of the PFRC were analyzed. The dynamic increase factor (DIF) of the dynamic compressive strength of the PFRC was also investigated. Finally, the damage dynamic constitutive model of PFRC was established, and the stress–strain curves obtained from the tests were fitted and analyzed.

## 2. Experimental Program

### 2.1. Raw Materials and Mixture Proportions

**Figure 2:**
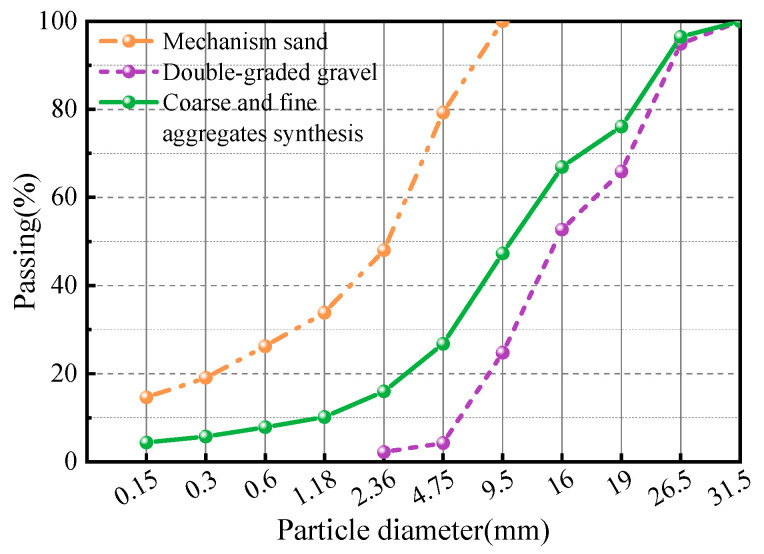
Granulometric curves for the fine and coarse aggregate.

**Figure 3:**
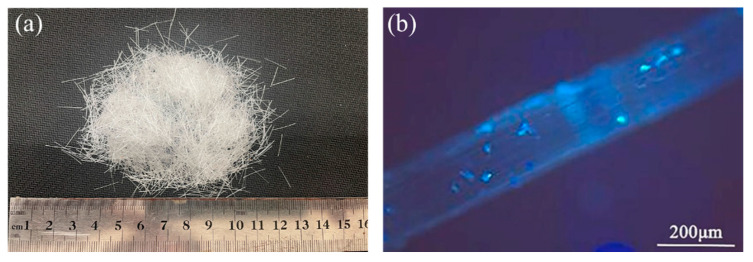
(**a**) Macro and (**b**) micro morphologies of POM fibers.

Ordinary Portland cement (P·O 42.5) was bought from Yunnan Hua Xin Cement, Co., Ltd. (Kunming, China); the material properties of the cement are shown in Table 1. F-type I low-calcium fly ash and S95-grade blast furnace slag powder were used to replace part of the cement; the material properties of fly ash and blast furnace slag powder are shown in Table 2 and Table 3, respectively. Coarse aggregates with 4.75–16 mm and 16–26.5 mm double-graded limestone gravel with an apparent synthetic density of 2748 kg/m^3^ were used. The fine aggregates were mechanism sand with an apparent density of 2708 kg/m^3^ and a fineness modulus of 3.13. The granulometric curves for the fine and coarse aggregate are shown in Figure 2. The external additive was a polycarboxylic acid high-performance water-reducing agent. The POM fibers were obtained from Chongqing Yuntianhua Tianjuxincai Co., Ltd. (Chongqing, China). Table 4 displays the fibers’ physical and mechanical properties. The macro and micro morphologies of POM fibers are shown in Figure 3, and the fiber micro morphology was observed using a ZEISS microscope (Carl Zeiss AG, Oberkochen, Baden-Württemberg, Germany). Table 5 displays the mix proportion of the OPC and PFRC, where PFRC-6, PFRC-8, PFRC-12, PFRC-16, and PFRC-24 denote POM fiber concrete with fiber lengths of 6, 8, 12, 16, and 24 mm, respectively.

### 2.2. Specimen Preparation

In order to ensure the quality and uniformity of the concrete specimens, the mixing process was strictly controlled. Firstly, the cementitious materials, coarse aggregates, and fine aggregates were dry-mixed for 1 min. Then, the fibers were slowly sprinkled in the mixture and dry-mixed for 3 min. Lastly, water and a water-reducing agent were added and we continued mixing until the fibers were dispersed evenly. Then, the fresh concrete was packed into a mold of 100 mm × 100 mm × 400 mm for vibration compaction and left to sit for 24 h in the chamber. The concrete was demolded and moved into a standard curing room (temperature was 20 ± 2 °C; relative humidity was more than 95%) for 28 d. In order to ensure the uniformity of the SHPB test specimens, the cuboid specimens with completed curing were core-drilled and cut. The surface of the specimens was polished to eliminate the influence of roughness on the test. The geometric size of the prepared specimens was ϕ75×40 mm, and the roughness of the end face was less than 0.05 mm, which conforms to the recommended value according to existing research results [11,12]. Before the SHPB test, the static compressive strength of OPC and PFRC was tested in this study. The test specimens were cubes of 150 mm × 150 mm × 150 mm, and the static compression test was carried out using a YE-200 hydraulic-type pressure tester with a loading rate of 20 KN/s. Each group was tested with three specimens, and the average static compressive strength is shown in Table 6.

### 2.3. SHPB Test

**Figure 4:**
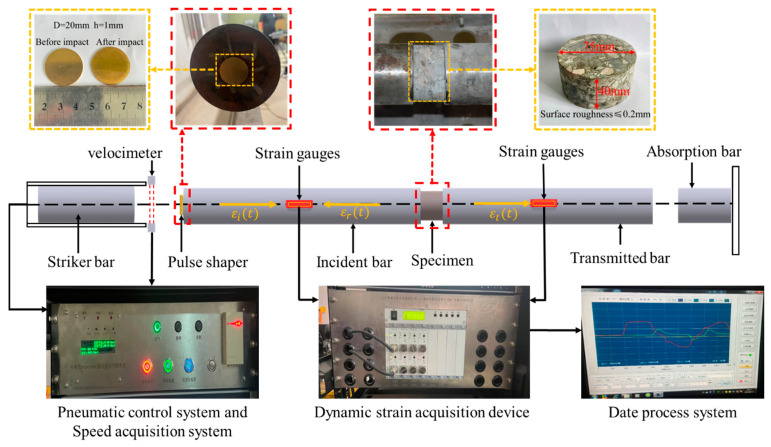
Schematic diagram of the SHPB test and the principle of stress wave propagation.

In this study, the dynamic compression mechanical properties of PFRC were studied with the ϕ80mm SHPB test setup, which is mainly composed of the loading system, the pressure bar system, and the data acquisition system. Figure 4 displays the schematic diagram of the SHPB test and the principle of stress wave propagation. When the striker bar impacts the incident bar, the incident wave $\varepsilon_{i}(t)$ is generated, and when the incident wave $\varepsilon_{i}(t)$ passes through the interface between the incident bar and the specimen, part of it is reflected to form the reflected wave $\varepsilon_{r}(t)$. The other part passes through the specimen to form the transmitted wave $\varepsilon_{t}(t)$ in the transmitted bar, where the incident wave $\varepsilon_{i}(t)$ and the reflected wave $\varepsilon_{r}(t)$ are measured by the strain gauges on the surface of the incident bar, and the transmitted wave $\varepsilon_{t}(t)$ is measured by the strain gauges on the surface of the transmitted bar. According to the stress wave propagation theory, the stress σ(t), strain ε(t), and strain rate $\dot{\varepsilon }(t)$ of the specimens can be obtained by processing the test data using the “three-wave method”, as shown in Equation (1).

(1) $$\left\{ \begin{array}{l} \sigma (t)=\frac{A_{0}E}{2A_{s}}\left[\varepsilon_{i}(t)+\varepsilon_{r}(t)+\varepsilon_{t}(t)\right]\\ \varepsilon (t)=\frac{C_{0}}{Ls}\int_{0}^{t}\left[\varepsilon_{i}(t)-\varepsilon_{r}(t)-\varepsilon_{t}(t)\right]\\ \dot{\varepsilon }(t)=\frac{C_{0}}{Ls}\left[\varepsilon_{i}(t)-\varepsilon_{r}(t)-\varepsilon_{t}(t)\right] \end{array}\right.$$

where E=206 GPa, $A_{0}=80\;\mathrm{mm}$, and $C_{0}=5139\;m/s$ are the elastic modulus, cross-sectional area, and elastic wave velocity of the bars, respectively. $A_{s}$ is the cross-sectional area of the specimens, and Ls is the original length of the specimens.

Concrete, as a brittle material, has a relatively small failure strain, and the rise time of the incident wave is short in conventional SHPB tests. In order to allow enough time for the specimen to reach stress equilibrium and to eliminate waveform oscillations, an H62 brass waveform shaper of ϕ20×1 mm in size was pasted in the center of the incident bar before the test. The waveform shaper is shown in Figure 4. In order to eliminate the influence of end friction confinement [13], medical Vaseline was evenly smeared on both surfaces of each specimen. Then, the specimen was located in the center between the incident bar and the transmitted bar. The strain rate of the SHPB test is closely related to the material characteristics of the tested specimen, and the test cannot accurately control the actual strain rate of the specimen [14]. In this study, the strain rate of the specimen was made approximately constant by controlling the same impact air pressure, and three levels of air pressure, 0.3 MPa, 0.5 MPa, and 0.7 MPa, were selected, and then three different strain rates of about 80.0 s^−1^, 110.0 s^−1^, and 150.0 s^−1^ were obtained by referring to the method of Hao et al. [15] regarding the determination of the strain rate. In order to ensure accurate and reliable test results, three parallel samples were taken from each batch in this test, and the average value was taken as the representative result.

## 3. Test Results and Discussion

### 3.1. SHPB Test Stress Uniformity Analysis

To ensure the validity of the test data, the SHPB test must satisfy two basic assumptions, namely, the one-dimensional elastic stress wave assumption and the stress uniformity assumption [11]. The one-dimensional elastic stress wave assumption is easily satisfied, and the validity of test data mainly depends on whether the specimen reaches a uniform state of stress before failure [16]. In this study, the validity of the test data was verified based on the stress uniformity assumption, and the transmitted waves at the incident surface of the specimen in the SHPB test were calculated according to Equation (2), which was used to check the stress uniformity.

(2) $$\varepsilon_{t}(t)=\varepsilon_{i}(t)+\varepsilon_{r}(t)$$

where $\varepsilon_{i}(t)$ is the incident wave, $\varepsilon_{r}(t)$ is the reflected wave, and $\varepsilon_{t}(t)$ is the transmitted wave.

**Figure 5:**
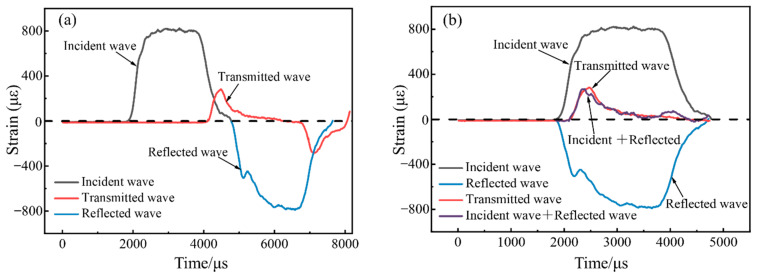
Typical stress wave: (**a**) before waveform alignment and (**b**) after waveform alignment.

Figure 5 shows the typical stress waves of the specimen under an impact loading. It can be seen that the waveforms of the incident waves, reflected waves and transmitted waves are less oscillating. The incident waves, reflected waves, and transmitted waves in Figure 5a are aligned at the wave head, and then the waveforms obtained by summing the incident waves and reflected waves can be compared with the waveforms of the transmitted waves to visually determine the stress uniformity, as shown in Figure 5b. It can be seen that the waveform obtained by adding the incident wave and the reflected wave agree well with the waveforms of the transmitted wave. This shows that the SHPB test performed in this study satisfies the stress uniformity assumption and that the test data are effective.

### 3.2. Failure Pattern

**Figure 6:**
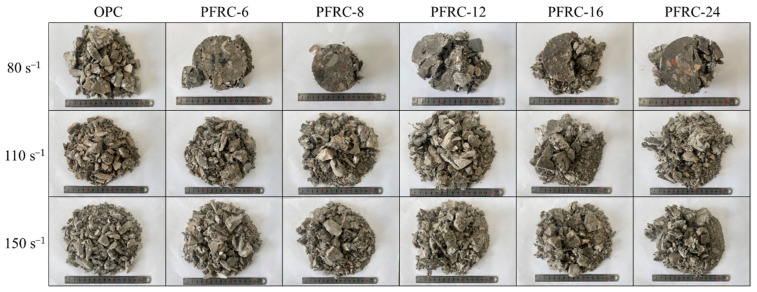
Failure patterns of specimens under different strain rates.

The typical failure pattern of each group of specimens at different strain rates is shown in Figure 6. It is obvious that with the increase in the strain rate, the number of medium and large pieces in each group of specimens after damage gradually decreased and the number of small pieces gradually increased. The degree of damage of the specimens increased with the increase in the strain rate.

It can also be seen from Figure 6 that when the strain rate was about 80.0 s^−1^, the failure pattern differed significantly between the OPC and PFRC. The OPC suffered brittle failure, and was broken into loose fragments. However, the central part of the PFRC was retained more completely, and the failure was dominated by edge cracking and falling off, with the PFRC-8 specimens being the most complete after failure. This indicates that when the strain rate is about 80.0 s^−1^, POM fiber helps maintain the integrity of the core area of the damaged specimen, reducing crack development, and POM fiber with a length of 8 mm has the best crack restraint ability. The OPC and PFRC specimens were cracked at strain rates of about 110.0 s^−1^ and 150.0 s^−1^, but the specimens of the PFRC had larger and more residual blocks after failure, and fibers connected the broken pieces. This indicates that the stress transfer mechanism between matrix and fibers still exists at high strain rates. POM fibers can significantly improve the impact crack resistance of concrete. This is because the fibers can form a fiber network in concrete, which can transfer the impact energy to the surrounding cement matrix under the impact load, causing the crush of the cement matrix, thereby creating some space inside the fiber network as a buffer zone, which can cushion the propagation of impact waves, thus improving the impact resistance of PFRC [17].

### 3.3. Dynamic Stress–Strain Curves

**Figure 7:**
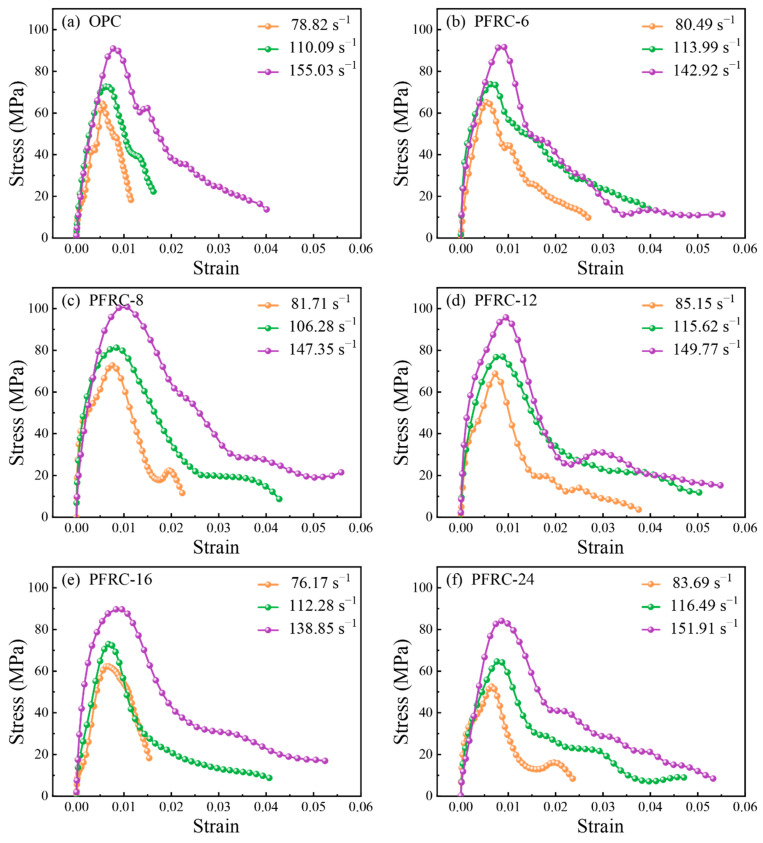
Stress–strain curves of specimens at different strain rates: (**a**) OPC; (**b**) PFRC-6; (**c**) PFRC-8; (**d**) PFRC-12; (**e**) PFRC-16; and (**f**) PFRC-24.

The dynamic stress–strain curve can comprehensively describe the mechanical behavior of concrete under impact loading. Figure 7 shows the average stress–strain curves of the OPC and PFRC at different strain rates. It can be seen that the stress–strain curves are similar in shape for each group of specimens, and all the curves can be divided into rising and falling sections. At the beginning of the rise, the compressive stress was low, the deformation of the specimen was mainly elastic deformation between the cement matrix and the aggregate, and the relationship between stress and strain was approximately linear. As the compressive stress increased, the microcrack gradually expanded, and the deformation of the specimen entered the elastoplastic stage. At this time, the slope of the stress–strain curve gradually reduced. When the stress reached the peak point, the specimen cracked and was damaged; the stress–strain curve started to drop, and the specimen showed strain-softening behavior [18].

It can also be seen in Figure 7 that the dynamic stress–strain curves of the OPC and PFRC were significantly affected by the strain rate; the higher the strain rate, the higher the peak stress, thus exhibiting the strain rate effect. Moreover, the rising strain intervals and falling strain intervals of the stress–strain curves of the PFRC were larger than those of the OPC, indicating that adding POM fibers of different lengths can improve the deformation capacity of concrete.

Table 7 shows the results of the dynamic compression mechanical properties of each group of specimens at different strain rates. The meanings of parameters in Table 7 are as follows: v is the striker bar velocity, $\bar{\dot{\varepsilon }}$ is the strain rate, $f_{c,d}$ is the dynamic compressive strength, $\varepsilon_{p}$ is the peak strain, $\varepsilon_{u}$ is the ultimate strain, DIF is the dynamic increase factor, $IT_{p}$ is the peak toughness, and $IT_{u}$ is the ultimate toughness.

### 3.4. Dynamic Compressive Strength Analysis

**Figure 8:**
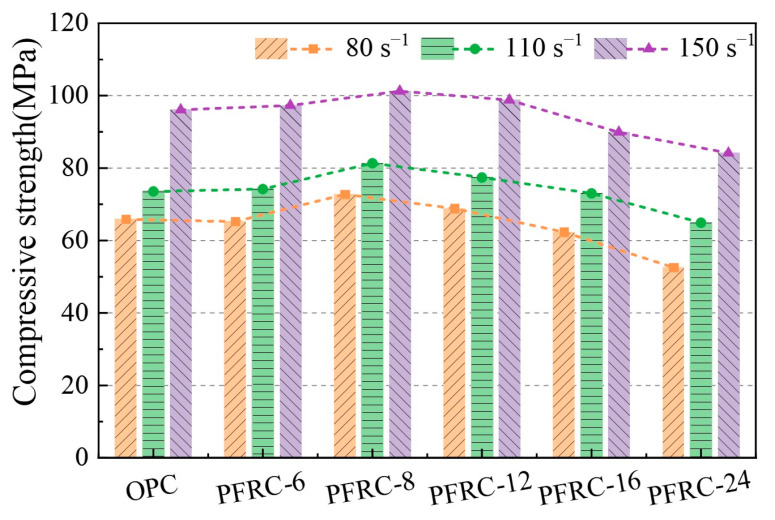
Dynamic compressive strength of each group of specimens at different strain rates.

**Figure 9:**
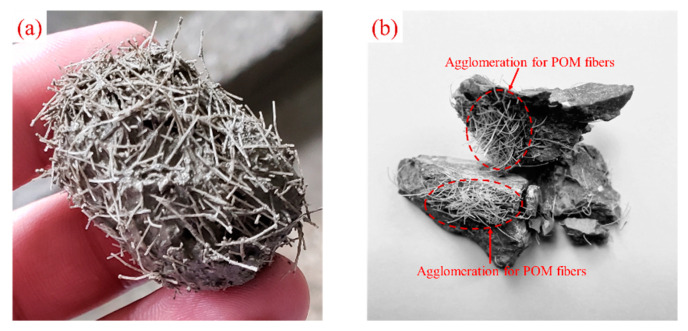
Agglomeration for POM 24 mm in length: (**a**) during the concrete mixing process and (**b**) after the specimens were broken.

The dynamic compressive strength is the stress value corresponding to the peak point of the stress–strain curve and is used to characterize the strength properties of the specimen under impact loading. The dynamic compressive strength of each group of specimens at different strain rates is shown in Figure 8. It can be seen that the dynamic compressive strength of PFRC-8 was optimal when the strain rates were about 80.0 s^−1^, 110.0 s^−1^, and 150.0 s^−1^, and, compared with the OPC, the dynamic compressive strength was increased by 10.4%, 10.6%, and 5.4%, respectively. However, compared with the OPC, the dynamic compressive strength of PFRC-6 and PFRC-12 did not increase significantly. This phenomenon may be because when the fiber content is the same, the number of POM fibers with a length of 6 mm is higher, which increases the degree of initial damage to the concrete. Their anchorage length is smaller and easy to pull out from the matrix, which has little improvement in the dynamic compressive strength of the concrete. POM fibers of length 12 mm have a smaller number of fibers working effectively on the failure surface of concrete and have insignificant improvement in the dynamic compressive strength of concrete. In contrast, the dynamic compressive strengths of both PFRC-16 and PFRC-24 decreased. The largest decrease was observed for PFRC-24, where the dynamic compressive strength decreased by 25%, 13%, and 14% at the three strain rates, respectively. With the increase in POM fiber length, the dynamic compressive strength of concrete tended to increase first and then decrease. The reason for this phenomenon is that when the fiber content is the same, shorter fibers are not conducive to the formation of a fiber network, and shorter fibers can lead to the reduction in anchorage length and the weakening ability to inhibit crack propagation [17,19]. When the fibers are too long, this leads to a decrease in the number of fibers that work effectively on the failure surface and they cannot effectively control the crack propagation [10]. It was also found in the test that when the fiber content was 0.9%, the POM fibers with a length of 24 mm appeared to agglomerate (Figure 9), which would increase the defects of the concrete matrix, which may also be the reason for the significant decrease in the dynamic compressive strength of PFRC-24.

An analysis of the results shown in Figure 8 also revealed that the dynamic compressive strength of the specimens in both the OPC and PFRC increased significantly with the increasing strain rate, with a significant strain rate effect, and the dynamic compressive strength was approximately linearly related to the strain rate, which is similar to the results of related studies on other types of fiber-reinforced concrete impact compression [15,20,21]. This “strain rate effect” may be caused by the transverse inertia effect of the specimen during the SHPB test [22,23], and can also be explained by the energy balance theory under a high-strain-rate dynamic load [6,24]: damage to concrete is caused by the initiation and propagation of cracks. In concrete, the energy required for crack initiation is higher than the energy required for crack propagation. Under impact loading, microcracks do not have enough time to expand before the specimen fails, and new cracks will be generated inside the specimen; thus, as the impact velocity increases, more new cracks will be generated, and thus more energy is required. There is not enough time for the concrete material to accumulate energy under impact loading, and energy can only be provided by increasing the stress. Therefore, the dynamic compressive strength of the concrete material increases with the strain rate.

### 3.5. Dynamic Increase Factor

In this study, the dynamic increase factor (DIF) was used to characterize the enhancement effect of dynamic loading on the compressive strength of concrete. The DIF is defined as the ratio of dynamic compressive strength to the static compressive strength of concrete, and the DIF values of OPC and PFRC are shown in Table 7. At present, based on the SHPB test, scholars have conducted extensive research on the DIF of concrete materials and proposed calculation models. Four representative DIF models were selected for comparison with the DIF results in this study.

CEB-fib model [25]:

(3) $$DIF=\left\{ \begin{array}{ll} {\left(\dot{\varepsilon }/{\dot{\varepsilon }}_{0}\right)}^{0.014} & \;\mathrm{for}\dot{\varepsilon }\leq 30s^{-1}\\ 0.012{\left(\dot{\varepsilon }/{\dot{\varepsilon }}_{0}\right)}^{1/3} & \;\mathrm{for}\dot{\varepsilon }>30s^{-1} \end{array}\right.$$

In Equation (3), $\dot{\varepsilon }$ and ${\dot{\varepsilon }}_{0}$ are the strain rate and quasi-static strain rate, respectively, and ${\dot{\varepsilon }}_{0}=30\times 10^{-6}$.

Tedesco and Ross [26]:

(4) $$DIF=\left\{ \begin{array}{l} 0.00965\log\dot{\varepsilon }+1.058\geq 1.0\;\;\;\;\;\;\mathrm{for}\dot{\varepsilon }\leq 63.1s^{-1}\\ 0.758\log\dot{\varepsilon }-0.289\leq 2.5\;\;\;\;\;\;\;\;\;\;\;\mathrm{for}\dot{\varepsilon }>63.1s^{-1} \end{array}\right.$$

Li and Meng [27]:

(5) $$DIF=\left\{ \begin{array}{l} 1+0.3438(\log\dot{\varepsilon }+3)\;\;\;\;\;\;\;\;\;\;\;\;\;\;\;\;\;\;\;\;\;\;\;\;\;\;\;\;\;\;\;\;\;\;\;\;\mathrm{for}\dot{\varepsilon }\leq 100s^{-1}\\ 1.792{\left(\log\dot{\varepsilon }\right)}^{2}-7.1372(\log\dot{\varepsilon })+8.5303\;\;\;\;\;\;\mathrm{for}\dot{\varepsilon }>100s^{-1} \end{array}\right.$$

Zhou and Hao [28]:

(6) $$DIF=\left\{ \begin{array}{l} 0.0225\log\dot{\varepsilon }+1.12\;\;\;\;\;\;\;\;\;\;\;\;\;\;\;\;\;\;\;\;\;\;\;\;\;\;\;\;\;\;\;\;\;\;\;\;\;\;\;\;\;\;\;\;\;\;\mathrm{for}\dot{\varepsilon }\leq 10.0s^{-1}\\ 0.2713{\left(\log\dot{\varepsilon }\right)}^{2}-0.3563(\log\dot{\varepsilon })+1.2275\;\;\;\;\;\;\;\;\;\;\;\mathrm{for}\dot{\varepsilon }>10.0s^{-1} \end{array}\right.$$

**Figure 10:**
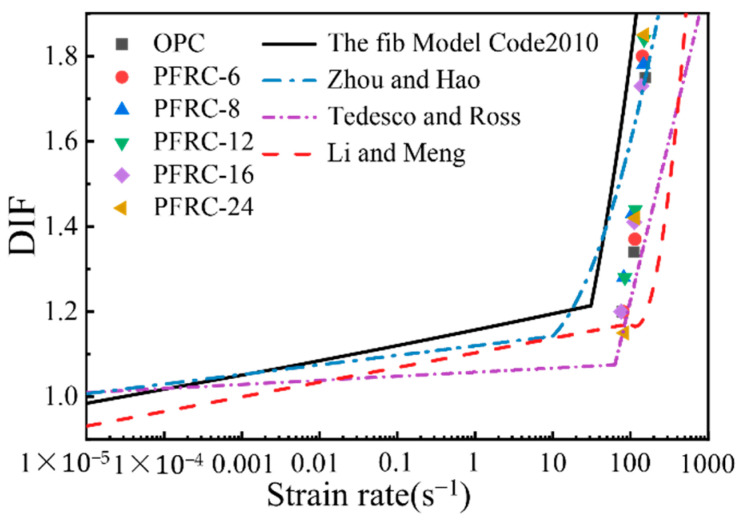
DIF for dynamic compressive strength of OPC and PFRC.

The DIF values of the OPC and PFRC and the four models are plotted in Figure 10. It can be seen that the effect of the strain rate on DIF was pronounced, and the DIF of the OPC and PFRC increased with the increase in strain rate, which is consistent with the trend in DIF variation in the four models. It is also found from Figure 10 that the amplitude of variation for DIF when the strain rate increased from about 80.0 s^−1^ to about 110.0 s^−1^ is smaller than the amplitude of variation when the strain rate increased from about 110.0 s^−1^ to about 150.0 s^−1^. When the strain rate increased from about 80.0 s^−1^ to about 150.0 s^−1^, the DIF of the OPC, PFRC-6, PFRC-8, PFRC-12, PFRC-16, and PFRC-24 increased by 45.8%, 50%, 39.0%, 43.8%, 44.2%, and 60.9%, respectively, and it was found that as the strain rate increased, PFRC-24 had the largest DIF growth, indicating that the DIF of PFRC-24 was the most sensitive to the strain rate. At present, the mechanism of the strain rate effect of DIF has not been fully explained. Li and Meng [27] and Cotsovos and Pavlovic [29] suggested that the increase in DIF with an increasing strain rate is mainly caused by lateral inertial restraint. However, Zhou and Hao [28] showed that lateral inertial restraint could be neglected when the strain rate is less than 200 s^−1^. Fu et al. [30] suggested that the “strain rate effect” is a combination effect produced by the action of crack propagation, the inertial effect, and the viscous effect of free water. The DIF may not be a material property, but a physical result generated by inertial restraint, microcrack propagation, specimen size, free water and other factors with the increase in the strain rate [31,32,33,34].

**Figure 11:**
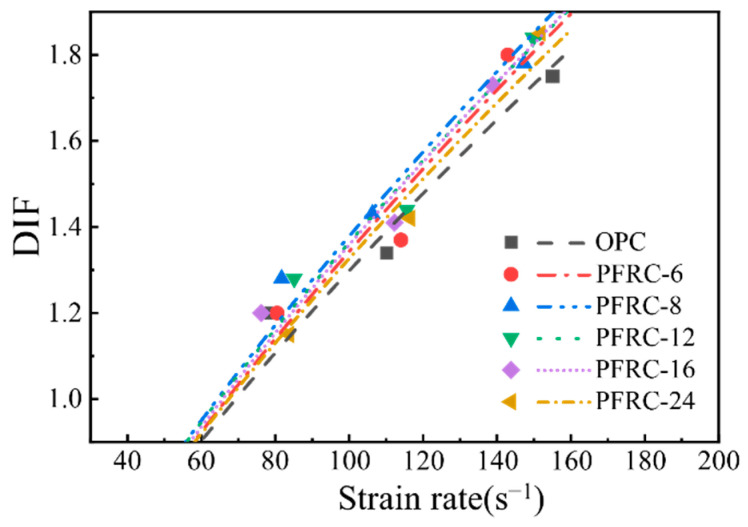
Empirical DIF relations for OPC and PFRC.

As can be seen from Figure 10, these four models overestimated or underestimated the DIF values of the POM fiber concrete and did not describe the evolution of the DIF of POM fiber concrete well. Different testing devices, specimen sizes, and different material compositions can lead to differences in the DIF models [35]; thus, to predict the DIF values of PFRC more accurately, the CEB-fib model [25] coefficients were modified by regression fitting of the test data; the results are shown in Table 8, and the corresponding empirical DIF relationships are shown in Figure 11. It can be seen that the POM fiber length variation has less effect on DIF. The variation trend in the DIF obtained from the test and the empirical relationship curve of the DIF obtained by the fitting is the same, and the variation in the strain rate significantly influences the DIF. In this study, the variation trends in DIF for OPC and PFRC with increasing strain rate were consistent with those reported previous research [23,36]. When the strain rate was within the range of this study, the fitted DIF empirical formulae R^2^ were all greater than 0.9, indicating that the fitted DIF empirical formulae are in good agreement with the experimental data and have high reliability, which can provide a theoretical basis for the study of dynamic reinforcement of concrete by POM fibers.

It can also be seen from Figure 10 that there is a transition strain rate for the variation in DIF with strain rate in all four models. That is, when the strain rate is below the transition strain rate, the DIF increases slowly with the increase in strain rate, and the material is low-strain-sensitive. When the strain rate exceeds the transition strain rate, the DIF increases sharply with increasing strain rate and the material changes to high strain sensitivity. The transition strain rate may also exist for POM-fiber-reinforced concrete. However, the lack of data for PFRC under quasi-dynamic loading made it difficult to accurately determine the transition strain rate for POM-fiber-reinforced concrete in this study. The DIF of POM-fiber-reinforced concrete under quasi-dynamic loading can be tested in the future to refine the DIF empirical formulae of POM-fiber-reinforced concrete and to determine its transition strain rate.

### 3.6. Dynamic Compression Deformation Analysis

Dynamic compression deformation is an important index for the analysis of deformation characteristics of specimens under impact loading. The dynamic compression deformation capacity of concrete materials can be expressed in terms of peak strain and ultimate strain. The peak strain of the concrete specimen is the strain value corresponding to the peak stress point of the dynamic stress–strain curve, and the dynamic ultimate strain is the maximum strain value of the dynamic stress–strain curve.

**Figure 12:**
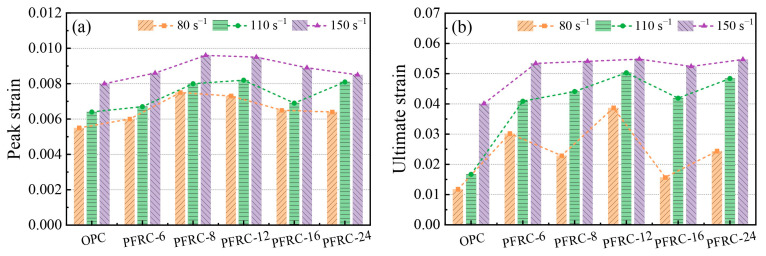
Dynamic compression deformation of each group of specimens at different strain rates: (**a**) dynamic peak strain and (**b**) dynamic ultimate strain.

The dynamic peak strain and dynamic ultimate strain of each group of specimens at different strain rates are shown in Figure 12a,b, respectively. It can be seen that at strain rates of about 80 s^−1^ and 150 s^−1^, the dynamic peak strain of PFRC showed a trend of increasing and then decreasing with the increase in fiber length, and the dynamic peak strain of PFRC-8 was the largest. The dynamic ultimate strain of PFRC-12 was maximum at strain rates of about 80 s^−1^, 110 s^−1^ and 150 s^−1^, respectively. Overall, the dynamic peak strain and dynamic ultimate strain of the PFRC were greater than those of the OPC at different strain rates. In the rising section of the stress–strain curve, the POM fibers could bridge the initial microcracks inside the concrete, which helped to slow down the degree of stress concentration at the crack tip and delayed the crack propagation, thus increasing the dynamic peak strain of the concrete. In the falling section of the stress–strain curve, the POM fiber bridged the cracks, and POM fiber delayed the development of cracks and slowed down the descent of stress, thus improving the ultimate strain of PFRC [37,38].

In Figure 12, it can also be seen that the dynamic peak strain and dynamic ultimate strain of each group of concrete specimens were significantly affected by the strain rate, and the higher the strain rate, the greater the dynamic peak strain and dynamic ultimate strain, which is consistent with the findings of Zhou et al. [39] and Zhang et al. [40]. This may be caused by the fact that as the strain rate increases, damage caused by a few macrocracks transforms into damage caused by multiple microcracks, resulting in an increase in cumulative strain [41]. However, the strain rate effects of the dynamic peak strain and dynamic ultimate strain are different from the strain rate effects of dynamic compressive strength characteristics, and there is no obvious linear correlation between dynamic peak strain and strain rate, or between dynamic ultimate strain and strain rate.

### 3.7. Impact Toughness Analysis

**Figure 13:**
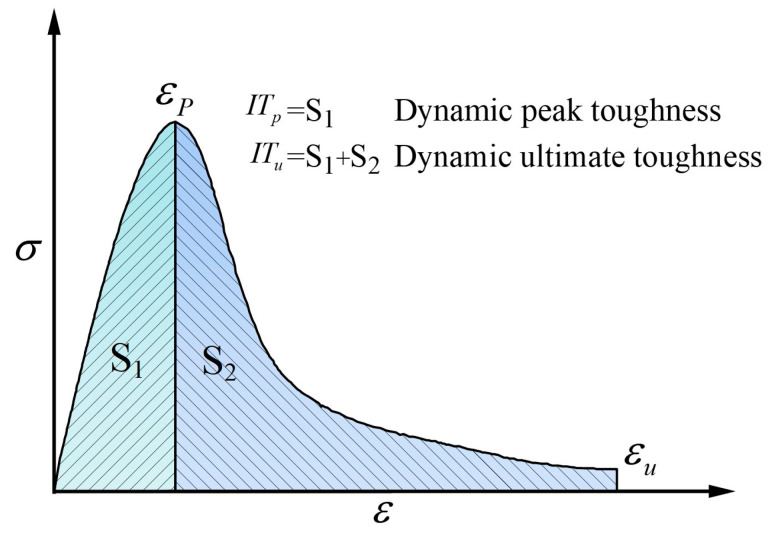
Schematic diagram of impact toughness calculation.

**Figure 14:**
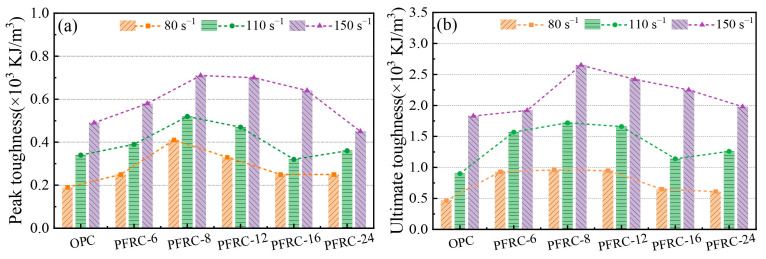
Impact toughness of specimens: (**a**) dynamic peak toughness and (**b**) dynamic ultimate toughness.

Impact toughness is an important index used to describe the dynamic mechanical properties of materials, and it is a comprehensive representation of material ductility and strength. Figure 13 shows a schematic diagram of the toughness calculation method, where $IT_{p}$ is the dynamic peak toughness, whose value is the integral area S1, and $IT_{u}$ is the dynamic ultimate toughness, whose value is the sum of integral area S1 and integral area S2. The dynamic peak toughness and dynamic ultimate toughness of each group of specimens at different strain rates are shown in Figure 14a,b. It can be seen that the dynamic peak toughness and dynamic ultimate toughness of the PFRC were higher than those of the OPC at different strain rates. This is due to the higher dynamic compression deformation capacity of the PFRC and its ability of POM fibers to retard crack expansion, which also requires additional energy consumption when being pulled out or fractured in the crack zone [24]. It can also be seen that the dynamic peak toughness and dynamic ultimate toughness of PFRC-8 were the largest. At strain rates of about 80.0 s^−1^, 110.0 s^−1^, and 150.0 s^−1^, the dynamic peak toughness of PFRC-8 increased by 115.8%, 52.9%, and 44.9%, respectively, and the dynamic ultimate toughness increased by 104.3%, 91.1%, and 44.8%, respectively, as compared to the OPC. The reason for this may be that when the content of POM fibers was the same, POM fibers with a length of 8 mm could ensure both a sufficient anchorage length and a sufficient number of effective fibers in the concrete specimens, which can effectively improve the impact resistance of the specimens and significantly enhance the impact toughness of the concrete.

It can also be seen from Figure 14 that both the dynamic peak toughness and dynamic ultimate toughness increased with the increasing strain rate, which is similar to the results of other studies on fiber-reinforced concrete [24,32,37]. This can be explained as follows: the higher the strain rate, the less time required for crack development. Before the microcrack grows into a macrocrack, more new cracks will be generated in the concrete specimen, and the crack formation requires more energy than the crack growth, which makes the dynamic peak toughness and dynamic ultimate toughness exhibit strain rate dependence [21]. In the dynamic compression experiments, the impact toughness of each group of specimens and the failure pattern of the specimens (Figure 6) were consistent, that is, with the increase in strain rate, the impact toughness of the specimens increased, more energy was absorbed, the specimens produced more microcracks and macrocracks, and they were more severely damaged.

## 4. Dynamic Constitutive Model of PFRC

### 4.1. Establishment of Damage Dynamic Constitutive Model

The dynamic stress–strain curves of POM-fiber-reinforced concrete obtained from the SHPB tests exhibited nonlinear viscoelastic characteristics. The nonlinear viscoelastic constitutive model (Z-W-T constitutive model) proposed by Zhu-Wang-Tang [42] can express the stress–strain relationship of concrete well. The Z-W-T constitutive model consists of a nonlinear elastomer and two viscoelastic Maxwell bodies with different relaxation times as the viscoelastic bodies, which can be expressed as follows:

(7) $$\sigma =f(\varepsilon )+\sigma_{m1}+\sigma_{m2}=E_{0}\varepsilon +\delta \varepsilon^{2}+\beta \varepsilon^{3}+E_{1}\int_{0}^{t}\dot{\varepsilon }\exp(-\frac{t-\tau }{\theta_{1}})d\tau +E_{2}\int_{0}^{t}\dot{\varepsilon }\exp(-\frac{t-\tau }{\theta_{2}})d\tau$$

where $f(\varepsilon )=E_{0}\varepsilon +\alpha \varepsilon^{2}+\beta \varepsilon^{3}$ represents the nonlinear elastic behavior of concrete; and $E_{0}$,α, and D are the elastic constants corresponding to the nonlinear elastic response. σ,ε, and $\dot{\varepsilon }$ are the stress, strain, and strain rate, respectively. $\sigma_{m1}$ and $\sigma_{m2}$ represent the viscoelastic behavior of concrete at low strain rates and high strain rates, respectively, where t and τ are the loading time and time variables. $E_{1}$ and $\theta_{1}$ are the elastic modulus and the relaxation time of low-frequency Maxwell bodies, respectively, $E_{2}$ and $\theta_{2}$ are the elastic modulus and the relaxation time of high-frequency Maxwell bodies, respectively.

From the dynamic stress–strain curve of the PFRC, it can be seen that the initial rising stage was approximately linear elastic, so the $\delta \varepsilon^{2}$ and $\beta \varepsilon^{3}$ in Equation (7) have an infinitesimal quantity that can be neglected [43,44]; thus, Equation (7) can be transformed into:

(8) $$\sigma =E_{0}\varepsilon +E_{1}\int_{0}^{t}\dot{\varepsilon }\exp(-\frac{t-\tau }{\theta_{1}})d\tau +E_{2}\int_{0}^{t}\dot{\varepsilon }\exp(-\frac{t-\tau }{\theta_{2}})d\tau$$

The mechanical properties of concrete are obviously affected by strain rate, which is more sensitive at high strain rates. The loading time of the concrete specimen under dynamic loading is $1∼10^{2}\mu s$, while the relaxation time is $10∼10^{2}s$, which leads to insufficient time for relaxation of the low-frequency Maxwell bodies [45]. The high-frequency Maxwell bodies can describe the viscoelastic mechanical behavior of the material at high strain rates. Therefore, the low-frequency Maxwell bodies in the Z-W-T constitutive model can be replaced by a simple linear elastomer, and Equation (8) can be converted to

(9) $$\sigma =E_{0}\varepsilon +E_{1}\varepsilon +E_{2}\int_{0}^{t}\dot{\varepsilon }\exp(-\frac{t-\tau }{\theta_{2}})d\tau =E_{a}\varepsilon +E_{2}\theta_{2}\dot{\varepsilon }[1-e^{-(\varepsilon /\theta_{2}\dot{\varepsilon })}]$$

The PFRC had a large number of random POM fibers inside and also contained a large number of pores and microcracks inside. Therefore, in order to make the constitutive model more representative, the damage factor D was introduced to modify the Z-W-T constitutive model of Equation (9) [46], and the generalized form of the constitutive model was obtained as follows:

(10) $$\sigma_{M}=\sigma (1-D)+Dk$$

where $\sigma_{M}$ is the modified stress, D is the damage factor, 0≤D≤1, and k is the load-bearing capacity of the POM fibers in the damaged zone.

Substituting Equation (9) into Equation (10), the modified Z-W-T constitutive model can be obtained as follows:

(11) $$\sigma_{M}=(1-D)\{E_{a}\varepsilon +E_{2}\theta_{2}\dot{\varepsilon }[1-e^{-(\varepsilon /\theta_{2}\dot{\varepsilon })}]\}+Dk$$

Concrete is a non-homogeneous brittle material with a low plastic strain, and the plastic deformation of concrete can be partially neglected. The damage to concrete is continuous during dynamic loading, and it can be assumed that the strength of each microunit obeys a probability distribution φ(ε) [47]; the damage factor D is related to φ(ε) as

(12) $$\frac{dD}{d\varepsilon }=\varphi (\varepsilon )$$

Assuming that the damage of the PFRC obeys the Weibull distribution [35,48], then

(13) $$\varphi (\varepsilon )=\frac{m}{\alpha }{\left(\varepsilon -\gamma \right)}^{m-1}\exp[-\frac{{\left(\varepsilon -\gamma \right)}^{m}}{\alpha }]$$

where m and α are the concrete material parameters and γ is the damage threshold. Based on Equations (12) and (13), the damage factor can then be obtained as

(14) $$D=\int_{\gamma }^{\varepsilon }\varphi (x)dx=\int_{\gamma }^{\varepsilon }\frac{m}{\alpha }{\left(x-\gamma \right)}^{m-1}\exp[-\frac{{\left(x-\gamma \right)}^{m}}{\alpha }]dx=1-\exp[-\frac{{\left(\varepsilon -\gamma \right)}^{m}}{\alpha }]$$

The cracks and pores inside the concrete material will cause it to have initial damage; therefore, the damage threshold γ=0, and the damage factor is further simplified as

(15) $$D=1-e^{\left(-\varepsilon^{m}/\alpha \right)}$$

Substituting Equation (15) into Equation (11), the damage dynamic constitutive model for PFRC can be obtained as Equation (16):

(16) $$\sigma_{M}=e^{\left(-\varepsilon^{m}/\alpha \right)}\{E_{a}\varepsilon +E_{2}\theta_{2}\dot{\varepsilon }[1-e^{-(\varepsilon /\theta_{2}\dot{\varepsilon })}]\}+[1-e^{\left(-\varepsilon^{m}/\alpha \right)}]k$$

In summary, the damage dynamic constitutive model of PFRC requires the fitting of six parameters, namely, m, α, $E_{a}$, $E_{2}$, $\theta_{2}$, and k. The parameters of the damage dynamic constitutive model for all the specimens at different strain rates are shown in Table 9.

### 4.2. Fitting Results and Analysis

From Table 9, it can be seen that the parameters m, $E_{a}$, $E_{2}$, and k increased with the increase in the strain rate; α and $\theta_{2}$ did not show a clear pattern. $E_{2}$ represents the dynamic modulus of elasticity of the PFRC, and the fitting results show that the dynamic modulus of elasticity of the PFRC was related to the strain rate and increased with the increase in the strain rate. k represents the bearing capacity of POM fibers, which indicates that the bearing capacity of the POM fibers increased with the increase in the strain rate.

**Figure 15:**
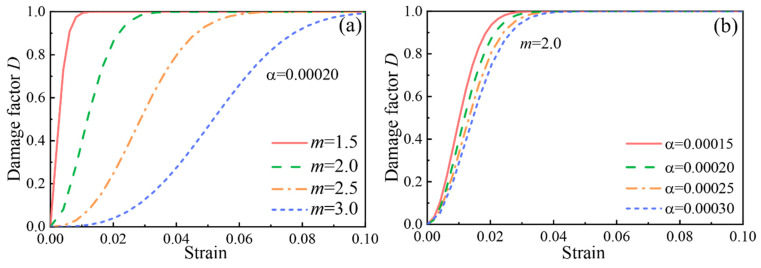
Influence of m and α on damage factors: (**a**) α as a fixed value and (**b**) m as a fixed value.

In the PFRC damage dynamic constitutive model, m and α are two important parameters used to evaluate the dynamic mechanical properties of PFRC. The influence laws of the changes in parameter S and parameter D on the dynamic damage factor when parameter A was chosen at a fixed value (0.0002) or with B at a fixed value (2.0) are shown in Figure 15a,b, respectively. It can be seen that the curve of the damage factor gradually flattened out as m increased when α was certain, and the curve of the damage factor gradually flattened out as α increased when m was certain, which indicates that the rate of damage evolution decreased as m and α increased. It can also be seen from Figure 15 that the variation in parameter m had a greater impact on the damage evolution as compared to the parameter α.

Based on the above analysis and combined with the results shown in Table 9, it was found that with the increase in the strain rate, parameter α increased, and the rate of damage evolution of the PFRC decreased. Several scholars have found that the damage evolution rate of basalt fiber concrete, polypropylene fiber concrete and ultra-early strength concrete also decreases with an increasing strain rate [6,35,49]. The reason for this phenomenon may be that at high strain rates, the cracks inside the PFRC do not have enough time to expand, resulting in a reduced rate of their damage evolution.

**Figure 16:**
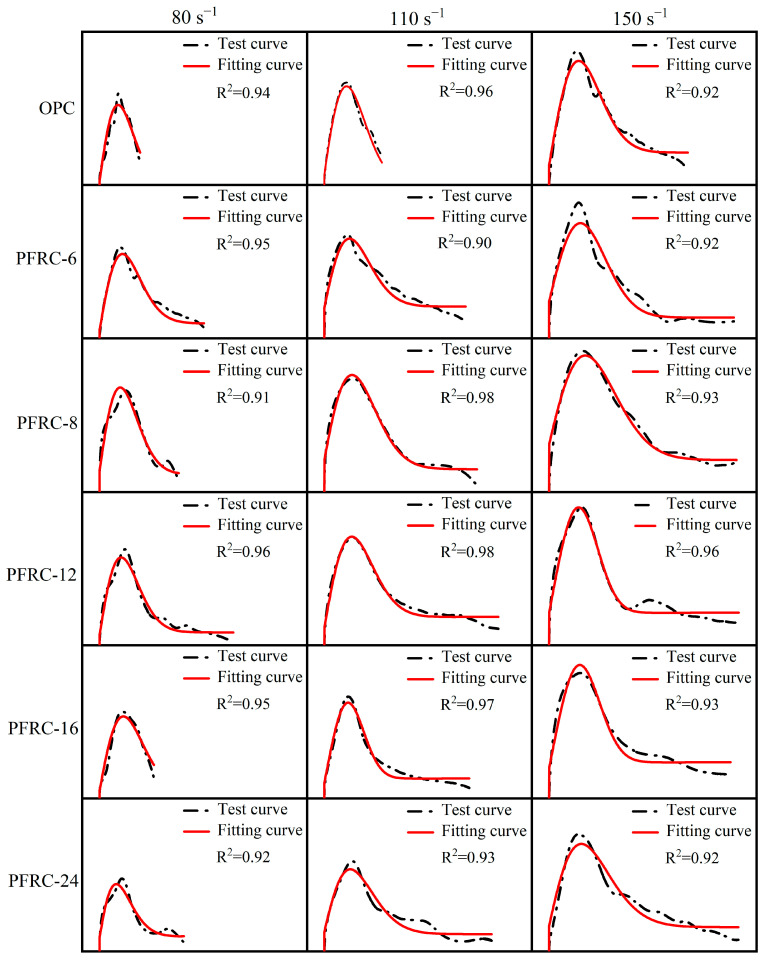
Comparison of experimental and fitted stress–strain curves of specimens at different strain rates.

According to the test data, the established PFRC damage dynamic constitutive model was fitted, and the test stress–strain curves and fitted stress–strain curves of each group of specimens at different strain rates are shown in Figure 16. It can be seen that the fitted stress–strain curves are in excellent agreement with the test stress–strain curves, which indicates that the established damage dynamic constitutive model can better describe the stress–strain relationship of PFRC at different strain rates. The constitutive model thus has reference value for the design of POM-fiber-reinforced concrete under dynamic impact loading.

## 5. Conclusions

In this study, the effects of strain rates on the dynamic compression mechanical properties of PFRC with different POM fiber lengths were studied using SHPB tests, and the damage dynamic constitutive model of PFRC was established based on the Z-W-T constitutive model. The main conclusions are as follows.

1. PFRC is a strain-rate-dependent material in which the dynamic compressive strength, dynamic increase factor, dynamic peak strain, dynamic ultimate strain, dynamic peak toughness, and dynamic ultimate toughness all increase with an increasing strain rate.
2. Different lengths of POM fibers can significantly improve the dynamic compressive deformation capacity and impact toughness of concrete. POM fibers with a length of 8 mm led to the greatest improvements in the dynamic compressive strength and impact toughness of the concrete.
3. The damage dynamic constitutive model of PFRC was established by optimizing the Z-W-T constitutive model with damage correction. The fitted curves of this model of PFRC are in good agreement with the experimental data of the PFRC, and the constitutive model can better describe the dynamic compressive mechanical properties of PFRC under different strain rate impact loading.
4. The constitutive model established can act as a reference for the design of PFRC under impact loading. For the PFRC studied in this paper, when the strain rate exceeded the range of 80~150 s^−1^, there were no test data to verify the validity of the model. In the future, the dynamic mechanical properties of PFRC under a wider range of strain rates can be studied, and the respective constitutive models can be improved.

## Figures and Tables

**Table 1:** Material properties of cement.

Specific Surface Area (m^2^/kg)	Specific Gravity	Setting Time (min)		Flexural Strength (MPa)		Compressive Strength (MPa)	
		Initial	Final	3d	28d	3d	28d
348	3.1	250	310	5.5	—	27.0	—

**Table 2:** Material properties of fly ash.

Density (kg/m^3^)	Fineness (%)	Ignition Loss (%)	Water Content (%)
2300	8.40	3.50	0.3

**Table 3:** Material properties of blast furnace slag powder.

Water Content (%)	Density (kg/m^3^)	Ignition Loss (%)	Activity Index (%)		Specific Surface Area (m^2^/kg)	Mobility Ratio (%)
			7d	28d		
0.40	2900	0.23	80	97	418	98

**Table 4:** Physical and mechanical properties of POM fiber.

Index	Diameter (mm)	Density (g/cm^3^)	Strength (MPa)	Elongation Rate (%)	Modulus (GPa)
Parameter	0.2	1.41	1000	15	8.5

**Table 5:** Mix proportions of OPC and PFRC.

NO.	W/B	POM Fiber Length (mm)	Dosage by Volume Fraction (%)	Mix Ratio (kg/m^3^)							
				Cement	Fly Ash	Slag	Water	Sand	Rough Stone (16–26.5 mm)	Fine Stone (4.75–16 mm)	Water Reducer
OPC	0.37	-	0	279.76	61.31	42.16	155.97	565.87	886.53	433.84	2.11
PFRC-6	0.37	6	0.9	279.76	61.31	42.16	155.97	565.87	886.53	433.84	2.11
PFRC-8	0.37	8	0.9	279.76	61.31	42.16	155.97	565.87	886.53	433.84	2.11
PFRC-12	0.37	12	0.9	279.76	61.31	42.16	155.97	565.87	886.53	433.84	2.11
PFRC-16	0.37	16	0.9	279.76	61.31	42.16	155.97	565.87	886.53	433.84	2.11
PFRC-24	0.37	24	0.9	279.76	61.31	42.16	155.97	565.87	886.53	433.84	2.11

**Table 6:** Static compressive strengths of PFRC.

Test No.	OPC	PFRC-6	PFRC-8	PFRC-12	PFRC-16	PFRC-24
Static compressive strength (MPa)	54.9	54.2	56.8	53.7	51.9	45.6

**Table 7:** Dynamic compression properties of OPC and PFRC.

Test No.	v (m/s)	$\bar{\dot{\varepsilon }}$ $\left(s^{-1}\right)$	$f_{c,d}$ (MPa)	$\varepsilon_{p}$	$\varepsilon_{u}$	DIF	$IT_{p}$ $\left(\times 10^{3}\;{\mathrm{kJ}/m}^{3}\right)$	$IT_{u}$ $\left(\times 10^{3}\;{\mathrm{kJ}/m}^{3}\right)$
OPC	4.21	78.82	65.89	0.0055	0.0118	1.20	0.19	0.47
	6.48	110.09	73.56	0.0064	0.0167	1.34	0.34	0.90
	8.18	155.03	96.08	0.0080	0.0401	1.75	0.49	1.83
PFRC-6	4.24	80.49	65.25	0.0060	0.0302	1.20	0.25	0.93
	6.45	113.99	74.23	0.0067	0.0409	1.37	0.39	1.57
	8.08	142.92	97.30	0.0086	0.0534	1.80	0.58	1.92
PFRC-8	4.27	81.71	72.77	0.0075	0.0229	1.28	0.41	0.96
	6.42	106.28	81.35	0.0080	0.0441	1.43	0.52	1.72
	8.04	147.35	101.27	0.0096	0.0541	1.78	0.71	2.65
PFRC-12	4.31	85.15	68.82	0.0073	0.0387	1.28	0.33	0.95
	6.55	115.62	77.41	0.0082	0.0503	1.44	0.47	1.66
	8.07	149.77	98.78	0.0095	0.0548	1.84	0.70	2.42
PFRC-16	4.17	76.17	62.35	0.0065	0.0157	1.20	0.25	0.65
	6.41	112.28	73.05	0.0069	0.0419	1.41	0.32	1.14
	8.04	138.85	89.92	0.0089	0.0524	1.73	0.64	2.25
PFRC-24	4.14	83.69	52.54	0.0064	0.0244	1.15	0.25	0.61
	6.43	116.49	64.93	0.0081	0.0484	1.42	0.36	1.26
	8.14	151.91	84.16	0.0085	0.0547	1.85	0.45	1.98

**Table 8:** Coefficients in the proposed DIF empirical formula for PFRC.

Test No.	$DIF=A{\left(\dot{\varepsilon }/{\dot{\varepsilon }}_{0}\right)}^{B}\;\mathrm{for}\dot{\varepsilon }>30s^{-1}$		
	A	B	$R^{2}$
OPC	$2.97\times 10^{-5}$	0.71	0.92
PRFC-6	$2.21\times 10^{-5}$	0.73	0.91
PRFC-8	$2.45\times 10^{-5}$	0.73	0.92
PRFC-12	$2.89\times 10^{-5}$	0.72	0.94
PRFC-16	$2.34\times 10^{-5}$	0.73	0.92
PRFC-24	$2.78\times 10^{-5}$	0.72	0.97

**Table 9:** Constitutive model fitting parameters.

Test No.	$\bar{\dot{\varepsilon }}$ $\;(s^{-1})$	m	α $\;(\times 10^{-4})$	$E_{a}$ (GPa)	$E_{2}$ (GPa)	$\theta_{2}$ $\;(\times 10^{-6})$	k	$R^{2}$
OPC	78.82	1.79	1.59	17.98	1.40	31	0	0.94
	110.09	1.80	2.16	17.48	2.32	30	0	0.96
	155.03	1.92	1.90	14.55	3.11	30	0	0.92
PFRC-6	80.49	1.77	2.32	14.46	2.30	30	10.66	0.95
	113.99	1.78	2.75	14.26	5.42	30	12.73	0.90
	142.92	2.05	1.49	10.25	6.64	28	14.87	0.92
PFRC-8	81.71	1.80	1.83	17.57	5.55	33	12.64	0.91
	106.28	1.82	2.95	13.33	7.20	30	16.40	0.98
	147.35	2.00	2.38	10.45	7.30	32	23.07	0.93
PFRC-12	85.15	1.78	2.07	15.69	2.81	29	9.23	0.96
	115.62	1.82	2.55	14.09	3.65	30	20.25	0.98
	149.77	2.50	0.17	13.27	5.197	30	23.29	0.96
PFRC-16	76.17	1.77	2.64	13.72	2.31	30	5.21	0.95
	112.28	2.33	0.20	12.85	3.03	33	14.27	0.97
	138.85	2.42	0.25	12.55	5.51	29	25.99	0.93
PFRC-24	83.69	1.57	3.57	15.18	3.90	30	10.75	0.92
	116.49	1.68	4.59	11.98	4.02	30	12.54	0.93
	151.91	1.68	6.58	11.44	4.10	30	17.59	0.92

## Data Availability

Not applicable.
